# The Use of JAK/STAT Inhibitors in Chronic Inflammatory Disorders

**DOI:** 10.3390/jcm12082865

**Published:** 2023-04-14

**Authors:** Giuseppina Caiazzo, Anna Caiazzo, Maddalena Napolitano, Matteo Megna, Luca Potestio, Luigi Fornaro, Melania Parisi, Maria Antonietta Luciano, Angelo Ruggiero, Anna Testa, Fabiana Castiglione, Cataldo Patruno, Maria Quaranta, Gabriella Fabbrocini

**Affiliations:** 1 Department of Clinical Medicine and Surgery, University of Naples Federico II, 80138 Naples, Italy; 2 Department of Medicine and Health Sciences Vincenzo Tiberio, University of Molise, 86100 Cambobasso, Italy; 3 Department of Health Sciences, University Magna Graecia of Catanzaro, 88100 Catanzaro, Italy

**Keywords:** JAK/STAT signaling pathway, JAK-STAT inhibitors, inflammatory disorders, atopic dermatitis, psoriasis, psoriatic arthritis, inflammatory bowel diseases

## Abstract

The Janus kinase (JAK)-signal transducer and activator of transcription (STAT) pathway plays a critical role in orchestrating immune and inflammatory responses, and it is essential for a wide range of cellular processes, including differentiation, cell growth, and apoptosis. Over the years, this pathway has been heavily investigated due to its key role in the pathogeneses of several chronic inflammatory conditions, e.g., psoriasis, atopic dermatitis (AD), and inflammatory bowel diseases (IBDs). Nevertheless, the impact of this pathway on the pathogenesis of inflammatory conditions remains unclear. This review describes the role of the JAK/STAT signaling pathway in the pathogenesis of inflammatory diseases such as psoriasis (Pso), psoriatic arthritis (PsA), AD, and IBD with a focus on ulcerative colitis (UC) and briefly resumes the use of JAK inhibitors in their clinical management.

## 1. JAK/STAT Signaling

**Figure 1:**
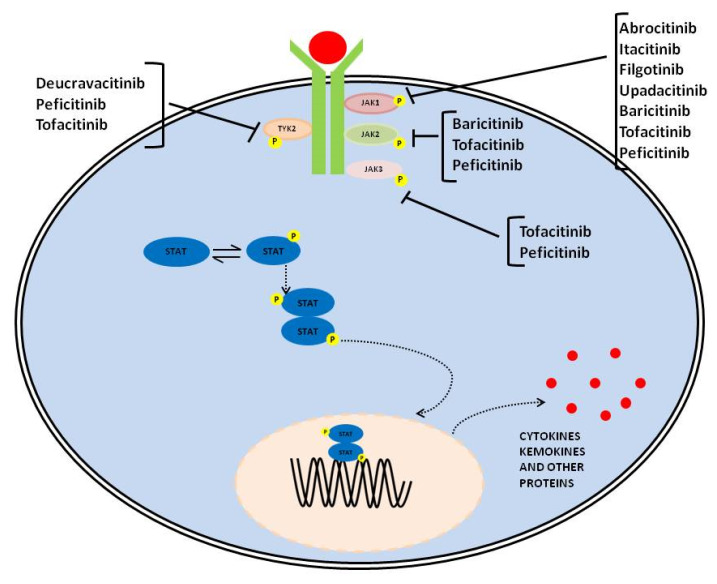
Graphical representation of JAK-STAT signaling pathway and therapeutic compounds suppressing it through the inhibition of one or several members of the family.

The JAK/STAT pathway is evolutionarily conserved, and it includes three main players: a ligand-receptor complex, for example, cytokines such as IL-2, IL-4, IL-6, IL-12, and IL-23; growth hormone (GH) growth factors (GF) and their receptors, one or two kinases belonging to the JAK family and one or two members of the STAT family [1,2,3]. Receptors are localized on the surface of specific cells and contain a binding site in the intracellular domain for tyrosine kinase JAK. The JAK family of kinase includes four members: JAK1, JAK2, JAK3, and tyrosine kinase 2 (TYk2) [4]. JAK1, JAK2, and TYk2 are expressed ubiquitously, whereas JAK3 is expressed mainly in hematopoietic cells [5]. JAK activation occurs upon ligand-mediated receptor multimerization, with two JAKs in close proximity, allowing trans-phosphorylation. The activated JAKs subsequently phosphorylate additional targets, including both the receptors and the major substrates, STATs [6]. Phosphorylated STATs dimerize and are translocated into the nucleus through the nuclear membrane to modulate the expression of target genes (Figure 1) [5,6]. STAT regulates transcription mechanisms in several ways: (1) by binding to its DNA target site to activate the transcription; (2) by forming a transcription complex with non-STAT transcription factors to induce the transcription mediated by STAT; (3) by its associations with non-STAT DNA-binding elements to promote STAT-dependent transcription; (4) by synergically activating the transcription through binding to clusters of independent DNA-binding sites.

The STAT family includes seven members, namely STAT1, STAT2, STAT3, STAT4, STAT5A, STAT5B, and STAT6 [4,7]. These members play different biological roles. In particular, STAT1 and STAT2 are central mediators of type I and III interferon signaling [6,7]; STAT3 is crucial for the differentiation of T helper (Th) 17; STAT4 functions in IL-12 signaling, and it is instrumental for the differentiation of Th 1 cells and, STAT6 is responsible for IL-4 and IL-13 signaling and, plays a key role in IgE-dependent allergic reactions [8,9]. Moreover, the biological function of JAK/STAT components in cytokine signaling has been highlighted by genetic knockout studies [9,10,11,12]. In vivo models have shown that *JAK1* or *JAK2* knockout mice die perinatally [10,11]; *JAK3*-, *TYK2*-, and *STAT6*-null mice suffer from immunodeficiency with a high susceptibility to infections [12,13], while mice lacking *STAT5A* or *STAT5b* are infertile and with a high rate of premature death due to severe anemia [14,15].

## 2. Methods

Search of the English-language literature regarding use of JAK-STAT inhibitors in inflammatory disease was carried out, in addition to JAK-STAT pathway. Different databases, namely PubMed, Embase, ResearchGate, Google Scholar, and Scopus, have been consulted using the following terms: JAK-STAT pathway, JAK-STAT inhibitors in psoriasis, psoriatic arthritis, ulcerative colitis, and atopic dermatitis. The clinical trials and preliminary results concerning investigational use of JAK-STAT inhibitors in inflammatory disease were searched on Clinicaltrial.gov (accessed on 4 April 2023). 

## 3. The Role of JAK-STAT Signaling Pathway in Psoriasis, Psoriatic Arthritis, Atopic Dermatitis, and Ulcerative Colitis

The involvement of JAK-STAT signaling pathway has been shown to play a key role in the pathogenesis of psoriasis, PsA, AD, and UC. Genetic studies and TYK2 deficient mouse models have indicated JAK/STAT signaling pathway to participate in the pathogenesis of psoriasis and PsA via activation of the IL-23/IL-17 axis and induction of keratinocytes (Kcs) and gamma–delta T cells proliferation. Activation of these cells is followed by an enhancement of pro-inflammatory cytokines and chemokines, i.e., IL-1β, IL-8, and CCL20 produced by KCs, ending in neutrophils recruitment and exacerbation of tissue damage [16].

In AD, dendritic cells (DCs) get activated by the thymic stromal lymphopoietin (TSLP) produced by KCs in a JAK1/JAK2-dependent manner [17]. Inflammatory cytokines produced by DCs and Kcs polarize CD4^+^ T cells towards the Th2 phenotype. This phenotype, through the production of cytokines, i.e., IL-4 and IL-13, is responsible for the isotype switch and IgE production in B cells acting through receptors with the common γ chain (γc) via the JAK1/JAK3 heterodimer [16].

In UC, pro-inflammatory cytokines such as IL-12, IL-23, and IL-6 play a key role in its pathogenesis via activation of JAK-STAT pathway. In particular, IL-12 and IL-23 activate STAT3 and STAT4 via JAK2 and TYK2, respectively, while IL-6 activates STAT3 via JAK1/2 and TYK2 [18,19].

## 4. JAK/STAT Signaling Pathway Inhibitors

Drugs acting on the JAK/STAT pathway have been designed to specifically inhibit several members of the family (e.g., tofacitinib, peficitinib–JAK1, JAK2, JAK3, and TYK2; baricitinib-JAK1 and JAK2) or one component (e.g., abrocitinib, itacitinib, filgotinib, upadacitinib–JAK1, and deucravacitinib–TYK2) through the mechanisms indicated (Table 1). Unlike biologics, these drugs can be orally administrated, showing no immunogenicity [20]. 

### 4.1. Tofacitinib

#### 4.1.1. Psoriatic Arthritis and Psoriasis

Tofacitinib is a JAK1 and JAK3 inhibitor, partially acting on JAK2 and TYK2 as well. JAK inhibitors interact with numerous cytokines involved in PsA pathogenesis, like common gamma chain-containing cytokines, interferon-γ, IL-12, as well as IL-6, 17, 22, and 23 [21]. The labeled dosage in PsA is 5 mg twice a day (b.i.d) per oral administration (os). Its efficacy has been demonstrated in a phase 3 trial in patients with PsA compared to tofacitinib 5 mg/die, adalimumab 40 mg every 2 weeks, or placebo. Particularly, at 3 months, the evaluated primary endpoint American College of Rheumatology (ACR) 20 response, was reached in 50% of subjects in the 5 mg tofacitinib group and 61% in the 10 mg tofacitinib group, compared with 33% in the placebo group. Notably, adalimumab achieved an ACR20 response in 52% of patients, being inferior to 61% of the 10 mg tofacitinib group [21]. Hence, tofacitinib (at both 5 mg or 10 mg) showed to be superior compared to placebo as regards ACR20 response at 3 months among PsA patients who failed or showed contraindication to synthetic disease-modifying anti-rheumatic drugs (DMARDs) [21]. 

In another phase 3 trial, PsA patients were randomized to receive tofacitinib 5 mg twice daily for 6 months and tofacitinib 10 mg twice daily for 6 months. At 3 months placebo subjects were blinded and switched to tofacitinib 5 mg or 10 mg twice daily. ACR20 response was achieved in 50% of tofacitinib 5 mg and 47% of tofacitinib 10 mg, compared with 24% of placebo (*p* < 0.001) [22]. In this trial, the tofacitinib efficacy of psoriatic skin lesions was evaluated as well. The tool used was the Psoriasis Area and Severity Index (PASI), which is an index used to express the severity of psoriasis. It combines the severity (erythema, induration, and desquamation) and percentage of the affected area.

Particularly, tofacitinib 10 mg, but not the 5 mg dose, was superior to placebo regarding the rate of PASI 75 response at 3 months. Particularly, PASI75 was reached in 42% for tofacitinib 10 mg, 20% for tofacitinib 5 mg, and 10% for placebo. The higher rate of PASI75 (42%) observed for tofacitinib 10 mg group with respect to the tofacitinib 5 mg was similar to efficacy results of two previous tofacitinib trials in patients with plaque psoriasis (PASI75 response was found in 15% of placebo, 43% tofacitinib 5 mg, 44% tofacitinib 10 mg, and 39% adalimumab) [21].

During the 3-month placebo-controlled period, adverse events (AEs) were reported to be higher in the tofacitinib 5 mg group (55%) and tofacitinib 10 mg (53%) compared to placebo (44%); the corresponding rates of serious AEs were 1%, 2%, and 2%, respectively. Globally, the most common AEs seen for all groups were upper respiratory tract infections. No deaths, gastrointestinal perforations, cancers, interstitial lung disease, or tuberculosis cases were reported. 

Two multi-site, randomized, double-blind clinical, phase 3 trials evaluated the efficacy and safety of 5 mg and 10 mg tofacitinib vs. placebo b.i.d in plaque psoriasis. In particular, at week 16, all placebo patients were re-randomized to tofacitinib 5 mg and followed up to 52 weeks. An achievement of PASI75 and Physician Global Assessment (PGA) 0/1 was evaluated at week 28. Patients that did not meet the scores were protocol-mandated to be withdrawn, with the option to be enrolled into an arm with 10 mg tofacitinib for 3 months. At week 28, a greater proportion of PASI75 was observed in the tofacitinib 10 mg group vs. tofafictinib 5 mg (68.8% vs. 55.6%, respectively). Among this class of subjects, results were maintained up to week 52 in 74.1% and 79.4%, respectively [22].

The most common AEs included serious infections, herpes zoster, opportunistic infections (e.g., tuberculosis), lung cancer, rare episodes of myocardial infarction, and cardiac arrest [23].

#### 4.1.2. Ulcerative Colitis

Tofacitinib was studied for the first time in UC in 2012 in a clinical trial of phase 2 [24] involving 194 adult patients with active UC who did not respond to standard treatment. The study period was 8 weeks, during which tofacitinib was given b.i.d. at doses of 0.5 mg, 3 mg, 10 mg, or 15 mg. Significant improvements of the primary outcomes (clinical response and remission, endoscopic response and remission) and secondary outcomes (value of C-reactive protein (CRP) and fecal calprotectin dosage) were observed in the 10 mg and 15 mg b.i.d. groups compared with placebo. This trial was followed by phase 3 clinical trials known as the OCTAVE program [25] composed by OCTAVE Induction 1 and 2, which were two randomized, double-blind, placebo-controlled, 8-week induction trials and by OCTAVE Sustain that was a maintenance study and consisted of one randomized, double-blind, placebo-controlled 52-week maintenance trial.

In the induction studies, UC patients received randomly tofacitinib 10 mg b.i.d. or placebo for 8 weeks as induction therapy. Overall, the patients previously treated with anti-tumor necrosis factor (anti-TNF) agents were 54%, and those exposed to oral prednisone were 46% at baseline. The primary endpoint was clinical remission at week 8. In the OCTAVE 1 induction study, the proportion of patients achieving clinical remission was higher in the tofacitinib 10 mg b.i.d. group (18.5% (*p* = 0.007)) compared with placebo (8.2% (*p* = 0.007)) including 598 patients in whom conventional therapy had failed while in the OCTAVE 2 induction trial that evaluated 541 patients, the primary endpoint occurred in 16.6% of patients in the tofacitinib arm vs. 3.6% in the placebo group (*p* = < 0.001). Concomitant therapy with corticosteroids did not influence efficacy rates at week 8, showing similar results in patients previously treated or not with anti-TNF drugs [25]. 

In the maintenance trial, 592 patients who were clinical responders after 8 weeks in the induction trials were randomly assigned 1:1:1 to receive a placebo, tofacitinib 5 mg, or 10 mg b.i.d. for 52 weeks. Clinical remission as primary endpoint often occurred in the 5 mg and 10 mg tofacitinib groups (34% and 47%, respectively) compared with placebo (11% (*p* < 0.001)) [25]. Mucosal healing as a secondary endpoint also was noted in a significantly larger proportion of patients in either treatment group (37%—5 mg and 46%—10 mg [*p* < 0.001]). Other secondary endpoints, such as clinical response, endoscopic remission, and Inflammatory Bowel Disease Questionnaire (IBDQ) remission, also improved importantly in both the tofacitinib group and placebo [25]. 

The safety profile of tofacitinib observed in these trials was acceptable and manageable. In the induction trials, the most common AEs observed were headache, nausea, nasopharyngitis, and joint pain mostly in the tofacitinib group 10 mg b.i.d compared to the placebo group [26]. 

Serious adverse events [SAEs], including pneumonia, anal abscess, and *Clostridium difficile* infection, were observed in 1.3% and 0.2%, respectively, in the OCTAVE 1 and OCTAVE 2 compared to the placebo group in which there were no serious infections. Moreover, herpes zoster (HZ) infection was reported in 0.6% (OCTAVE 1) and 0.5% (OCTAVE 2) of the patients receiving tofacitinib than the placebo group. In the maintenance studies, HZ infection was found more frequently in the 10 mg group than the other two groups, with a statistically significant difference [26,27]. 

An interesting study conducted by Sandborn WJ et al. reported that an increase in dosage of tofacitinib is related to the risk of herpes zoster (HZ) infection [28]; in particular, there is a dose-dependent association between the use of tofacitinib and this risk [29]. To reduce the risk of infection in these patients, *Colombel* [30] proposed that the vaccination with the live HZ vaccine should be considered in all patients before starting treatment. Immunization with this type of vaccine should be performed at least two weeks before the start of therapy because it is not possible to administer at the same time the live vaccines and tofacitinib. In the most immunosuppressed patients, the new inactivated vaccine for recombinant HZ virus (Shingrix^®^) introduced in 2017 can be administered [30]. 

Another study evaluated the incidence of opportunistic infections (excluding HZ), reporting a very low rate of these infections (16%) with only four cases of cytomegalovirus colitis, pulmonary cryptococcosis, histoplasmosis, and cytomegalovirus hepatitis [28,31]. 

Another important observation in these trials was the impairment of serum lipid profile, including total cholesterol (TC), low-density lipoprotein cholesterol (LDL), and high-density lipoprotein cholesterol (HDL) [25]. A dose-dependent increase of LDL and HDL was observed at week 8 of treatment. These parameters became normal several weeks after stopping tofacitinib. Therefore, lipid levels monitoring should be done 4–8 weeks after the start of therapy [32].

The use of tofacitinib in all patients with risk factors for venous thromboembolism (VTE), both deep vein thrombosis (DVT) and pulmonary embolism (PE) [33] such as prior VTE, heart failure, cancer, immobilization, use of combined hormonal contraceptives or hormone replacement therapy and congenital bleeding disorders should be limited [34]. An interesting study conducted by Sandborn WJ et al. [35] reported the incidence of DVT and PE in the tofacitinib UC program, including 1157 patients in the overall cohort. It showed that one patient had DVT and four had PE receiving 10 mg of tofacitinib twice daily and with venous thromboembolism risk factors. However, all patients treated with tofacitinib, in particular high-risk patients, should be particularly supervised when VTE risk is assessed [34,36].

There are few data on tofacitinib use during pregnancy. Mahadevan et al. [37] reported that the frequencies of spontaneous abortions and congenital malformations were similar to those observed for the general population with the risk factors.
Drug’s package insert.**JAK Inhibitor****Clinical Trials****Condition****Therapeutic Dose****Side Effects****EMA/FDA Indications**TofacitinibOPAL Broaden OCTAVE PsA UC5 mg b.i.d. 10 mg b.i.d for 8 weeks followed by 5 mg b.i.d.-Infections -lung cancer -rare episodes of myocardial infarction -impairment of serum lipid profile -venous thromboembolismApproved Approved

### 4.2. Peficitinib

#### Ulcerative Colitis

Peficitinib is an oral JAK inhibitor that inhibits all four JAK isoforms showing a higher selectivity for JAK3. In a phase IIb study, the effects of peficitinib were evaluated in moderately to severely active UC. This trial is a placebo-controlled, double-blind, randomized trial including 219 patients [38]. They were randomly assigned to different groups receiving placebo or peficitinib at a dose of 25 mg, 75 mg, or 150 mg once daily, or 75 mg peficitinib b.i.d. Efficacy of treatment after 8 weeks was the primary endpoint. Clinical response, clinical remission, mucosal healing, change from baseline in the Inflammatory Bowel Disease Questionnaire (IBDQ), and normal values of inflammatory markers at week 8 were the secondary endpoint. 

For the first endpoint, no statistically significant differences among groups were found at week 8. For all secondary endpoints, peficitinib was more efficacious in a greater proportion of patients receiving a dose of 75 mg or higher once daily. However, the C-reactive protein (CRP) was not consistently reduced after the treatment, but calprotectin normalization was seen at week 8 in more patients receiving the drug at a dose ≥75 mg once daily compared with a placebo [38]. 

Serious adverse events were infrequent; the most common reported infection was nasopharyngitis. Moreover, only one patient treated with peficitinib 75 mg b.i.d had a positive result on the Clostridium difficile stool test [38].
Drug’s package insert.**JAK Inhibitor****Clinical Trials****Condition****Therapeutic Dose****Side Effects****EMA/FDA Indications**PeficitinibASP015KUC75 mg/150 mg dailynasopharyngitis -positive result of Clostridium difficile stool testDiscontinued

### 4.3. Baricitinib

#### Atopic Dermatitis

Baricitinib is a selective JAK1 and JAK2 inhibitor. A phase II study enrolling 124 patients randomized to receive baricitinib 4 mg (*n* = 38), baricitinib 2 mg (*n* = 37), or placebo (*n* = 49) for 16 weeks showed a statistically significant higher percentage of patients achieving Eczema Area and Severity Index 50 (EASI 50) response in baricitinib 4 mg at week 16 compared with placebo (61% vs. 37%, *p* = 0.027). EASI is a validated scoring system that grades the physical signs of atopic dermatitis/eczema. The EASI assessment integrates body surface and the intensity of lesional skin into one composite score. EASI is the core outcome for measuring the clinical signs of eczema in all trials. This statistically significant result was not assessed in the baricitinib 2 mg cohort. Globally, five patients in the placebo group, one in the baricitinib 2 mg cohort, and five in the baricitinib 4 mg group discontinued the study for AEs [39]. 

Two independent multicenter, double-blind, phase III studies (BREEZE-AD1 and BREEZE-AD2) enrolling adult patients (*n* = 624 in BREEZE-AD1 and *n* = 615 in BREEZE-AD2) affected by moderate-to-severe AD randomized (2:1:1:1) to daily receive placebo, baricitinib 1 mg, 2 mg, or 4 mg for 16 weeks showed that more patients reached a Validated Investigator’s Global Assessment of AD (vIGA-AD) of 0 (clear) and 1 (almost clear) in the baricitinib cohorts compared with placebo in BREEZE-AD1 and BREEZE-AD2 [40]. Of note, itch improvement was achieved as early as week 1 for 4 mg and week 2 for 2 mg baricitinib groups. No serious AEs were reported [40]. A long-term analysis of up to 68 weeks of responders or partial responders (patients achieving a vIGA-AD of 0, 1, or 2) in BREEZE-AD1 and BREEZE-AD2 was performed in the BREEZE-AD3 study.

The proportion of the responder/partial responder patients treated with baricitinib 4 mg (*n* = 70), reaching vIGA-AD (0,1) at week 16 (BREEZE-AD3 baseline) and at week 68 was 45.7% and 47.1%, respectively. Among the responder/partial responder baricitinib 2 mg population (*n* = 54), 46.3% at week 16 and 59.3% at week 68 reached validated investigator global assessment (vIGA)-AD (0, 1) [41]. BREEZE-AD5 assessed the efficacy and safety of baricitinib monotherapy on 440 American adults affected with moderate-to-severe AD unresponsive to topical treatments, randomized (1:1:1) to receive placebo and baricitinib 1 mg and 2 mg daily. At week 16, a higher proportion of patients achieving EASI75 was reported in patients receiving baricitinib 2 mg, 1 mg, and placebo (30%, 13%, 8%; *p* < 0.001 for 2 mg vs. placebo). Safety results were similar to those of other baricitinib AD studies [42]. BREEZE-AD7 was a phase III randomized (1:1:1), placebo-controlled study investigating the impact of baricitinib 4 mg, 2 mg, or placebo plus topical corticosteroids (TCS) on health-related quality of life (HRQoL) and Dermatology Life Quality Index (DLQI). The latter is a ten-question questionnaire used to measure the impact of the evaluated skin condition on the patient’s quality of life. A total of 329 patients were enrolled. A statistically significant improvement in DLQI starting at week 2 was reported in the baricitinib 4 mg and 2 mg cohort compared with placebo (baricitinib 4 mg, *p* ≤ 0.001; baricitinib 2 mg, *p* ≤ 0.05); improvements were maintained up to week 16 for baricitinib 4 mg. As regards the effectiveness, a vIGA-AD score of 0 or 1 was reached by 34 (31%), 26 (24%), and 16 (15%) patients treated with baricitinib 4 mg, baricitinib 2 mg, and placebo (*p* = 0.004 for baricitinib 4 mg group; *p* = 0.08 for the baricitinib 2 mg group) [43].
Drug’s package insert.**JAK Inhibitor****Clinical Trials****Condition****Therapeutic Dose****Side Effects****EMA/FDA Indications**BaricitinibBREEZEAD4 mg dailynasopharyngitis -headache -HZ infectionApproved

### 4.4. Abrocitinib

#### Atopic Dermatitis

Abrocitinib is an oral JAK1 selective inhibitor. Its safety and efficacy were assessed in patients affected by moderate to severe AD in a randomized, double-blinded, placebo-controlled phase IIb trial. Patients were randomized (1:1:1:1:1) to receive abrocitinib 10 mg, 30 mg, 100 mg, 200 mg, or placebo four times a day for 12 weeks. At week 12, an investigator’s Global Assessment (IGA) of “clear” or “almost clear” was reported in 43.8%, 29.6%, 8.9%, 10.9%, and 5.8% of patients receiving 200 mg, 100 mg, 30 mg and 10 mg of abrocitinib and placebo, with a statistical significance in abrocitinib 200 mg and 100 mg cohort compared with placebo. Similar results were observed in terms of EASI reduction, with an improvement of 82.6%, 59.0%, and 40.7% in patients treated with 200 mg, 100 mg, and 30 mg of abrocitinib. However, only EASI reduction in patients treated with 200 and 100 mg of abrocitinib was statistically significant compared to placebo. 

At week 12, a statistically significant reduction in peak pruritus numerical rating scale (P-NRS) score was observed in the 200 mg (–25.4%; *p* = 0.003) and 100 mg (−20.7%; *p* = 0.02) group compared with placebo. Regarding safety, 44 (16.5%) patients discontinued treatment for AEs. In particular, two patients reported serious AEs treatment-related: one patient in the 200 mg group and one patient in the 100 mg group developed pneumonia and eczema herpeticum, respectively [44].

JADE MONO-1 was a multicenter, randomized phase III study enrolling patients with moderate-to-severe AD receiving abrocitinib 100 mg (*n* = 135), abrocitinib 200 mg (*n* = 137), and placebo (*n* = 61) four times a day (QID) for 12 weeks. An IGA response of “clear” or “almost clear” was achieved by 43.8%, 23.7%, and 7.9% of patients receiving abrocitinib 200 mg, abrocitinib 100 mg, and placebo, respectively, with a significant reduction compared to placebo in abrocitinib 100 mg (15.8%, *p* = 0.0037) and 200 mg (36.0%, *p* < 0.0001) group. EASI75 was reached by 62.7%, 39.7%, and 11.8% of patients receiving abrocitinib 200 mg, abrocitinib 100 mg, and placebo, respectively. A statistically significant improvement in the P-NRS score was reported (57.2% and 37.7% of patients receiving 200 mg and 100 mg of abrocitinib, respectively, vs. 15.3% of the placebo group), with an effective response already observed at week 2 in abrocitinib treated patients. Serious AEs, including asthma, IBD, dehydration, and peri-tonsillitis, were reported in five (3.2%) patients receiving abrocitinib 200 mg; appendicitis, seizures, dizziness, and acute pancreatitis in five (3.2%) patients in abrocitinib 100 mg group; and worsening of AD, appendicitis, and meniscal degeneration in three (3.9%) patients of the placebo group. Discontinuation rates of 6% due to gastrointestinal disorders and vomiting were reported for both 200 mg and 100 mg abrocitinib AD and IBD groups. A higher discontinuation rate of 9.1% compared to the one observed for the abrocitinib cohorts was reported [45]. The effectiveness and safety of abrocitinib were also reported in another multicenter, double-blind, randomized phase III study (JADE MONO-2), enrolling 391 patients affected by moderate-to-severe AD, receiving abrocitinib 200 mg (*n* = 155), abrocitinib 100 mg (*n* = 158), or placebo (*n* = 78) QID for 12 weeks. At week 12, a statistically significant higher percentage of patients receiving abrocitinib 200 mg and 100 mg reached an IGA of “clear” or “almost clear” compared with placebo (38.1% and 28.4% vs. 9.1%; *p* < 0.001) as well as EASI-75 (61.0% and 44.5% vs. 10.4%; *p* < 0.001). Similarly, a greater proportion of patients achieved a P-NRS reduction of at least four points at week 12 in abrocitinib 200 mg and 100 mg cohort compared with placebo (55.3% and 45.2% vs. 11.5%; *p* < 0.001).

Regarding safety, a case of herpangina and a case of pneumonia was reported in abrocitinib 100 mg cohort, leading to treatment discontinuation. Globally, 5 (3.2%) patients in the abrocitinib 200 mg group, 6 (3.8%) in the abrocitinib 100 mg cohort, and 10 (12.8%) in the placebo group discontinued treatment for AEs, mainly for headache and AD worsening [46].

The maintenance of abrocitinib-induced response with continuous abrocitinib treatment was assessed in a phase III study (JADE REGIMEN). A total of 798 patients with moderate-to-severe AD responding to abrocitinib 200 mg monotherapy for 12 weeks were randomized (1:1:1) to receive abrocitinib 200 mg, abrocitinib 100 mg, or placebo for 40 weeks. Patients experiencing a worsening of AD received rescue treatment (abrocitinib 200 mg plus topical therapy). The probability of flair during maintenance dose was 18.9%, 42.6%, and 80.9% in the abrocitinib 200 mg, abrocitinib 100 mg, and placebo groups. Moreover, 36.6%, 58.8%, and 81.6% of patients experiencing flare regained an IGA of “clear” or “almost clear” in the abrocitinib 200 mg, abrocitinib 100 mg, and placebo groups. AEs were reported in 63.2% and 54.0% of patients during maintenance with abrocitinib 200 and 100 mg, respectively [47].

Abrocitinib 100 mg and 200 mg once a day were compared to placebo at 12 weeks and to dupilumab at 2 weeks in the JADE COMPARE trial. A total of 838 patients were randomized to receive abrocitinib 200 mg (*n* = 226), 100 mg (*n* = 238), 300 mg dupilumab (*n* = 243), and placebo (*n* = 131). At week 12, an IGA response of “clear” or “almost clear” was observed in 48.4%, 36.6%, 36.5%, and 14.0% of patients in the 200 mg, 100 mg abrocitinib group,300 mg dupilumab group, and placebo group. Similarly, an EASI75 response at week 12 was observed in 70.3%, 58.7%, 58.1%, and 27.1% in respective groups. Only abrocitinib 200 mg dosage showed to be superior to dupilumab with respect to itch response at week 2. No statistically significant differences were observed in abrocitinib and dupilumab cohorts at week 16 [48].

Finally, JADE TEEN, a randomized (1:1:1), double-blind, placebo-controlled, parallel-group, phase 3 trial, evaluated the effectiveness and safety of abrocitinib (200 mg or 100 mg once a day) plus topical treatment vs. placebo plus topical treatment in adolescent patients (aged 12–18 years) affected by moderate to severe AD. A total of 285 adolescent patients were enrolled. At week 12, a statistically significant higher percentage of patients receiving abrocitinib (200 mg or 100 mg) vs. placebo reached an IGA response of “clear” or “almost clear” (46.2%; 41.6% vs. 24.5%; *p* < 0.05 for both), EASI75 response (72.0%; 68.5% vs. 41.5%; *p* < 0.05 for both), and P-NRS reduction of at least 4 (55.4%; 52.6% vs. 29.8%; *p* < 0.01 for 200 mg vs. placebo). Serious AEs were reported in abrocitinib 200 mg group (*n* = 1) and placebo (*n* = 2) [49].
Drug’s package insert.**JAK Inhibitor****Clinical Trials****Condition****Therapeutic Dose****Side Effects****EMA/FDA Indications**AbrocitinibJADE-MONOAD200 mg daily-nausea -nasopharyngitis -headache -respiratory infectionApproved

### 4.5. Itacitinib

#### Ulcerative Colitis

Another oral, selective JAK1 inhibitor is itacitinib, which is being evaluated in a phase 2, double-blind, placebo-controlled trial for induction and maintenance treatment in patients with active ulcerative colitis. It has already been investigated in different trials for the therapy of some medical conditions such as endometrial cancer, melanoma, and B-cell malignancies [50].
Drug’s package insert.**JAK Inhibitor****Clinical Trials****Condition****Therapeutic Dose****Side Effects****EMA/FDA Indications**ItacitinibPhase I/II—INCB052793UC35 mg dailynasopharyngitis -headache -nauseaPhase II

### 4.6. Filgotinib

#### 4.6.1. Psoriatic Arthritis

Filgotinib is an oral, selective inhibitor of JAK1 being tested for different inflammatory disorders, such as PsA, rheumatoid arthritis, ankylosing spondylitis, and UC. Filgotinib is able to inhibit the action of numerous inflammatory cytokines and chemokines (e.g., s IL-6, CXCL10, IL-23, IL-22, IL-12, ICAM-1 ) involved in PsA pathogenesis.

A phase 2 trial evaluated the role of filgotinib in PsA, randomizing patients to filgotinib 200 mg daily or placebo for 16 weeks. ACR20 was observed in 52 (80%) of the filgotinib group and 22 (33%) of placebo at week 16. AEs were not different between the placebo group (57%) and the filgotinib group (59%). The most common AEs were nasopharyngitis: 12% for the placebo group and 15% for filgotinib group [51]. In another multicenter, double-blind, placebo-controlled, phase 2 study, randomized subjects with active PsA received filgotinib 200 mg daily or placebo for 16 weeks. Compared with placebo, filgotinib significantly improved HRQoL in patients with active PsA, as measured by psoriasis arthritis impact of disease (PsAID9) [52].

#### 4.6.2. Ulcerative Colitis

The efficacy of filgotinib for the treatment of UC was assessed in a double-blind, randomized, placebo-controlled phase 2b/3 study, also known as a SELECTION trial [53]. It was composed of two induction studies (induction studies A and B) and one maintenance study. The induction studies have enrolled patients who had an incomplete clinical response, intolerance, or loss of response to any TNF antagonist, such as infliximab, adalimumab, and golimumab or vedolizumab. The main difference between induction studies A and B was the previous exposure to biological therapy. In study A, biologically naive patients (*n* = 659) were included, while study B evaluated failed biological therapy patients (*n*= 689). For each induction study, these patients were randomized 2:2:1 into three groups to receive filgotinib 200 mg, filgotinib 100 mg, or placebo once daily for 10 weeks. Clinical remission at week 10 was the primary endpoint defined as Mayo endoscopic subscore ≤ 1, rectal bleeding subscore = 0, and ≥1-point decrease in stool frequency subscore from baseline to obtain a subscore ≤ 1.

At week 10, 64 biologic-naïve patients (26.1%) receiving filgotinib 200 mg met clinical remission compared with placebo (15.3%) in induction study A, while in induction study B, the primary endpoint occurred in 30 biologic-experienced patients (11.5%) compared to 6 patients receiving placebo (4.2%). There were no statistically significant differences in clinical remission in the filgotinib 100 mg group and in the placebo group. At week 11, patients who met clinical remission or clinical response were accessed in the maintenance study encompassing 664 patients. These patients were randomly assigned 2:1 into the induction filgotinib dose or the placebo groups. Patients previously receiving placebo as induction stayed on it. Similarly to the primary endpoint, week 58 was used as a time point for clinical remission.

A significantly higher proportion of patients given filgotinib met the primary endpoint than placebo (37.2%—200 mg group vs. 11.2%—placebo group, *p* < 0.0001; 23.8%—100 mg group vs. 13.5%—placebo group, *p* = 0.0420). In addition, key secondary endpoints, including endoscopic remission, histological remission, and Mayo Clinic score (MCS) remission, which is one of the most used disease activity tools based on rectal bleeding, stool frequency, physician assessment, and endoscopy appearance, were also measured. In particular, at week 10, secondary endpoints were met in a greater proportion of biologic treatment-naïve patients (24.5% (*p* = 0.0053), 12.2% (*p* = 0.0047), and 35.1% (*p* < 0.0001), respectively) and biologic-experienced patients (9.5%, 3.4% and 19.8% (*p* > 0.05), respectively) given filgotinib 200 mg once daily compared with placebo. However, there were no significant differences in the secondary endpoints between the filgotinib 100 mg group and the placebo group at week 10. In the maintenance study, a significantly higher proportion of MCS remission, endoscopic and histological was detected in the filgotinib 200 mg arm than to placebo group (34.7% vs. 9.2%, *p* < 0.0001; 15.6% vs. 6.1%, *p* < 0.025; 38.2% vs. 13.3%, *p* < 0.025, respectively) and also similarly in the filgotinib 100 mg arm compared with placebo (22.7% vs. 13.5%, *p* = 0.0658; 13.4% vs. 7.9%, *p* > 0.05; 27.9% vs. 18.0%, *p* > 0.05, respectively). The most common AEs reported in the induction studies were headache and nasopharyngitis: 28 (5.0%) at a dose of 100 mg, 22 (4.3%) at a dose of 200 mg group, and 13 (4.7%) in the placebo arm. Interestingly, four patients reported HZ infection: one receiving filgotinib 100 mg (0.2%) and three given filgotinib 200 mg (0.6%); malignancies (excluding non-melanoma skin cancer) occurred in one patient (0.2%) of both filgotinib groups respectively while only one patient treated with filgotinib 200 mg developed pulmonary embolism (0.2%). In the maintenance study, the most frequent AEs were headache, arthralgia, nasopharyngitis, abdominal pain, upper respiratory tract infections, and worsening of ulcerative colitis: 8 (4.5%) at a dose of 100 mg and 7 (7.7%) in the respective placebo group; 9 (4.5%) at a dose of 200 mg and no patients in the respective placebo group. HZ infection occurred only in one patient receiving filgotinib 200 mg (0.5%); no pulmonary embolism events were reported [53,54].
Drug’s package insert.**JAK Inhibitor****Clinical Trials****Condition****Therapeutic Dose****Side Effects****EMA/FDA Indications**FilgotinibEQUATOR SELECTIONPsA UC200 mg daily 200 mg daily-nasopharyngitis -headache -HZ infectionPhaseII Approved

### 4.7. Upadacitinib

#### 4.7.1. Psoriatic Arthritis

Upadacitinib is an oral, reversible, selective JAK1 inhibitor approved for the treatment of PsA at the dosage of 15 mg daily. Its efficacy was demonstrated in a phase III trial compared to adalimumab, where subjects were randomized to receive oral upadacitinib at a dose of either 15 mg or 30 mg daily, 40 mg subcutaneous adalimumab every other week, or placebo followed by 15 mg or 30 mg upadacitinib daily (1:1 ratio) starting at week 28 and up to 56 weeks. At week 12, ACR20 was observed in 70.6% of subjects receiving 15 mg dose of upadacitinib, 78.5% in the upadacitinib 30 mg group, 36.2% for receiving a placebo, and in 65.0% in the adalimumab group [55]. In all treatment groups, the percentage of patients achieving ACR20/50/70 was maintained from week 24 to 56, with a higher percentage of patients originally randomized to upadacitinib 15 mg and 30 mg achieving ACR20/50/70 compared with adalimumab at week 56 (74.7% vs. 68.5% for upadacitnib 15 or 30 mg and adalimumab, respectively). In line with results through week 24, ACR20/50/70, PASI75/90/100, and minimal disease activity responses were maintained with upadacitinib through week 56 and were generally numerically higher than with adalimumab; inhibition of radiographic progression was also maintained. AEs such as an increase of blood creatine phosphokinase and upper part respiratory infection were more common with upadacitinib 30 mg versus upadacitinib 15 mg and adalimumab [55]. In the phase 3 SELECT-PsA 2 study, patients were randomized to 56 weeks of blinded treatment with oral upadacitinib 15 or 30 mg once daily or placebo switched to upadacitinib 15 or 30 mg once daily at week 24. Clinical efficacy endpoints assessed through week 56 included the proportion of patients achieving ACR20/50/70; PASI75 and PASI 90/100% improvement (PASI90/100; among patients with BSA > 3% body surface area of psoriasis at baseline) [56]. At week 56, as regard musculoskeletal outcomes, the ACR20/50/70 were achieved respectively from 59.7/40.8/24.2% of patients with upadacitinib 15 mg, and from 59.2/38.5/26.6% with upadacitinib 30 mg. At week 56, patients who switched to upadacitinib from placebo showed clinical responses similar to patients who received upadacitinib from baseline. Furthermore, as regard skin outcomes, the PASI75/90/100 were achieved respectively from 52.3/40.8/26.9% of patients with upadacitinib 15 mg and from 58.8/47.3/35.1% with upadacitinib 30 mg. The nasopharyngitis and upper respiratory tract infection were the most frequently AEs signaled [57].

#### 4.7.2. Ulcerative Colitis

Upadacitinib was evaluated in a phase 2, double-blind, placebo-controlled, dose-ranging, randomized trial known as the U-ACHIEVE study [58] to assess its efficacy for the treatment of active ulcerative colitis. The U-ACHIEVE program was composed of three trials: a dose-ranging induction study of phase 2b, a dose-confirming induction study of phase 3, and a maintenance study of phase 3. In the first study, 250 patients were randomly assigned to different groups receiving a placebo or four several doses of sustained-release formulations of upadacitinib (7.5 mg, 15 mg, 30 mg, or 45 mg) once daily for 8 weeks. Results showed that clinical remission rate was met for doses of 15 mg once daily or higher (8.5% at dose of 7.5 mg (*p* = 0.052), 14.3% at dose of 15 mg (*p* = 0.013), 13.5% at dose of 30 mg (*p* = 0.011) and 19.6% at dose of 45 mg (*p* = 0.002) once daily). A larger proportion of patients also met endoscopic improvement (endoscopic sub-score ≤ 1) with upadacitinib. Histological remission (described as a Geboes score less than 2) was significantly met in patients exposed to upadacitinib than in patients taking a placebo [58]. The second induction study confirmed these results. In the maintenance study, clinical remission occurred significantly more in patients receiving upadacitinib (15 mg 63 of 148; 30 mg 80 of 154) than those receiving placebo (18 of 149). The most frequently reported AEs were nasopharyngitis, creatine phosphokinase elevation, and acne.

#### 4.7.3. Atopic Dermatitis

A phase 2b dose-ranging study enrolling 167 patients affected by moderate-to-severe AD undergoing treatment with upadacitinib 7.5 mg (*n* = 42), 15 mg (*n* = 42), 30 mg (*n* = 42), or placebo (*n* = 41) for 16 weeks showed dose-dependent results in terms of effectiveness. Indeed, EASI75, EASI90, and IGA 0/1 responses were achieved by 48%, 26%, and 29% of patients receiving upadacitinib 15 mg QID. Upadacitinib 30 mg QID showed a 20% greater efficacy for these endpoints relative to 15 mg QID [59]. Measure Up 1 and Measure Up 2 were two multicenter, randomized, double-blind, placebo-controlled, phase 3 studies investigating the effectiveness and safety of upadacitinib in adolescents and adults affected by moderate-to-severe AD. Patients were randomly assigned (1:1:1) to receive upadacitinib 15 mg, upadacitinib 30 mg, or placebo once daily for 16 weeks. In particular, 285, 281, and 281 patients received upadacitinib 30 mg, upadacitinib 15 mg, or placebo in Measure Up 1 trial, whereas 282, 276, and 278 were treated with upadacitinib 30 mg, upadacitinib 15 mg or placebo in the Measure Up 2 study. At week 16, a statistically significant higher proportion of patients achieving EASI75 response was assessed in the upadacitinib 30 mg cohort (80% in Measure Up 1, 73% in Measure Up 2), upadacitinib 15 mg cohort (70% in Measure Up 1, 60% in Measure Up 2) compared with placebo (16% in Measure Up 1, 13% in Measure Up 2, *p* < 0.0001 for both). Similarly, the proportion of patients who achieved a vIGA-AD response of 0/1 was significantly greater in the upadacitinib 30 mg (62% in Measure Up 1, 52% in Measure Up 2), and in the upadacitinib 15 mg cohort (48% in MeasurUp 1, 39% in Measure Up 2), compared with placebo (8% in Measure Up 1, 5% in Measure Up 2, *p* < 0.0001 for both). The incidence of serious AEs was similar among groups [60]. The effectiveness and safety of upadacitinib plus topical corticosteroids compared with placebo in moderate-to-severe AD management were evaluated in a randomized, double-blind, placebo-controlled, phase 3 study (AD Up). A total of 901 patients were randomized to receive upadacitinib 30 mg plus topical corticosteroids (*n* = 297), upadacitinib 15 mg plus topical corticosteroids (*n* = 300), or placebo plus topical corticosteroids (*n* = 304). At week 16, a statistically significant higher percentage of patients achieving EASI75 response was reported in the upadacitinib 30 mg plus topical corticosteroids and upadacitinib 15 mg plus topical corticosteroids groups compared with placebo (77% and 65% vs. 26%, *p* < 0.0001 for both doses). Similarly, 59%, 40%, and 11% of patients in the upadacitinib 30 mg plus topical corticosteroid group, upadacitinib 15 mg plus topical corticosteroid group, and placebo plus topical corticosteroid group achieved a vIGA-AD response of 0/1 at week 16 (*p* < 0.0001 for both doses compared with placebo). The incidence of the discontinuation of treatment due to AEs was similar among treatment groups [61]. An extension up to week 52 of the AD Up study showed that the efficacy for upadacitinib 30 mg plus topical corticosteroids and upadacitinib 15 mg plus topical corticosteroids reached at week 16 were maintained through week 52, demonstrating long-term maintenance of effectiveness and a favorable safety profile of upadacitinib plus topical corticosteroids in patients with moderate-to-severe AD [62]. The effectiveness and safety of upadacitinib in adult patients with moderate-to-severe AD were also evaluated in a 24-weeks head-to-head study with dupilumab (Heads Up). In particular, 348 subjects were randomized to receive upadacitinib 30 mg QID, and 344 were randomized to receive dupilumab at the labeled dosage. At week 16, 71.0% and 61.1% of patients receiving upadacitinib and dupilumab reached an EASI75 response, respectively (*p* = 0.006). Moreover, upadacitinib showed superiority vs. dupilumab in improvement in Worst Pruritus NRS as early as week 1 (31.4% vs. 8.8%; *p* < 0.001), achievement of EASI75 as early as week 2 (43.7% vs. 17.4%; *p* < 0.001), and achievement of EASI100 at week 16 (27.9% vs. 26 7.6%; *p* < 0.001). With regard to safety, serious infection, eczema herpeticum, HZ, and laboratory-related AEs were more frequent in patients undergoing upadacitinib, while rates of conjunctivitis and injection-site reactions were higher for patients who received dupilumab [63]. Promising results have also been reported in real-life [64].
Drug’s package insert.**JAK Inhibitor****Clinical Trials****Condition****Therapeutic Dose****Side Effects****EMA/FDA Indications**UpadacitinibSELECT-PsA U-ACHIEVE MEASURE UPPsA UC AD15 mg daily 45 mg daily for 8 weeks, followed by 15/30 mg daily 15/30 mg daily-nasopharyngitis -upper respiratory tract infection -an increase in blood creatine phosphokinaseApproved Approved Approved

### 4.8. Deucravacitinib

#### Psoriasis and Psoriatic Arthritis

Deucravacitinib, an oral, selective TYK2 inhibitor, acts by binding to the enzyme’s regulatory domain. This unique binding mechanism provides high functional selectivity for TYK2. Indeed, a phase 2 trial showed Deucravacitinib selectivity in inhibiting TYK2, compared to other JAK-STAT inhibitors in vitro [65]. A phase 2 trial evaluated the drug in plaque psoriasis, randomizing them to receive deucravacitinib orally at a dose of 3 mg every other day, 3 mg daily, 3 mg twice daily, 6 mg twice daily, or 12 mg daily or to receive a placebo. At week 12, the percentage of patients with a 75% or greater reduction in the PASI score was 7% with placebo, 9% with 3 mg every other day (*p* = 0.49), 39% with 3 mg daily (*p* < 0.001), 69% with 3 mg twice daily (*p* < 0.001), 67% with 6 mg twice daily (*p* < 0.001), and 75% with 12 mg daily (*p* < 0.001). The most frequent AEs were nasopharyngitis (placebo 4%, 3 mg every other day 1%, 3 mg daily 9%, 3 mg twice daily 11%, 6 mg twice daily 16%, 12 mg daily 5%). Five serious AEs were reported in four patients: two events in one placebo group patient (hemorrhagic anemia and hemorrhoidal hemorrhage); gastroenteritis due to rotavirus in one patient receiving 3 mg every other day; accidental eye injury in one patient receiving 3 mg daily; dizziness due to vestibular dysfunction with a history of the same in one patient receiving 3 mg twice daily. In addition, there was one case of in situ malignant melanoma (stage 0) diagnosed at day 96 after the first dose of deucravacitinib 3 mg daily [66]. Moreover, the efficacy of deucravacitinib in psoriasis was confirmed in another phase 2 trial where subjects were randomized to receive for 12 weeks oral placebo or 3 mg every other day, 3 mg QD, 3 mg BID, 6 mg BID, or 12 mg QD. Mean percentage change from baseline in absolute PASI score as well as the proportion of patients achieving BSA < 1% and < 3% (26.7% (3 mg BID), 37.8% (6 mg BID), 38.6% (12 mg QD)) and (51.1% (3 mg BID), 44.4% (6 mg BID), and 56.8% (12 mg QD)) respectively, in the in deucravacitinib treatment groups was greater than the placebo group at each time point through Week 12. No data regarding safety were reported [67]. On the other hand, deucravacitinib (6 mg or 12 mg or placebo once a day for 16 weeks) was evaluated for the treatment of PsA in a phase 2 trial. ACR-20 response was significantly higher with deucravacitinib 6 mg once a day (52.9%) and 12 mg once a day (62.7%) versus placebo (31.8%) at week 16. AEs such as nasopharyngitis, upper respiratory tract infection, sinusitis, bronchitis, rash, diarrhea, headache, and acne were observed at a higher frequency at both deucravacitinib doses compared with placebo [68].
Drug’s package insert.**JAK Inhibitor****Clinical Trials****Condition****Therapeutic Dose****Side Effects****EMA/FDA Indications**DeucravacitinibNCT03881059PSO PsA6 mg daily-nasopharyngitis -upper respiratory tract infection -sinusitis, bronchitis -diarrhea -headache, acneApproved Phase II

## 5. JAK/STAT Signaling Pathway Inhibitors: News on Safety

Changes in the recommendation on the clinical use of JAK inhibitors (Table 2), such as tofacitinib, upadacitinib, baricitinib, abrocitinib, and filgotinib, have been made by the European Medicines Agency (EMA) following a warning from the US FDA in 2021 and the post-marketing ORAL Surveillance study, which showed an increased risk of onset of serious side effects in older rheumatoid arthritis patients with cardiovascular risk factors treated with tofacitinib when compared to anti-TNF therapy [69]. In particular, the EMA advises that JAK inhibitors should be recommended only when no suitable alternative therapies are present for patients with cancer and or cardiovascular risk factors and patients older than 65 years.

## 6. Conclusions and Future Perspectives

After the era of biologics, the development of JAK/STAT inhibitors and their successful use in inflammatory diseases heralds an exciting new chapter in therapeutic management. In fact, JAK/STAT inhibitors have shown powerful clinical efficacy in several inflammatory diseases, including AD, Pso, PsA, and UC, for which they have been licensed in the USA and in Europe. From our perspective, it is important to highlight that the JAK/STAT inhibitors are not all the same. They block different JAK subunits showing peculiarities in the action mechanism. Moreover, based on their different ability to inhibit and their broad action, these drugs can contribute to the onset of adverse events, some of which are expected and some of which are more enigmatic. In general, the most common are upper respiratory tract infections, HZ infections, urinary tract infections; diarrhea, increased blood creatine phosphokinase; headache, etc.

Therefore, in order to better reduce the onset of side effects, there is a need to draw a treatment selection algorithm that would help clinicians in their clinical practice. Moreover, additional efforts should also be invested in the identification of putative prognostic biomarkers that would help clinicians in patients’ therapeutic switch management. In conclusion, in light of the reviewed data, the patient’s safety remains an open challenge that needs to be further enriched with real-life experience.

## Figures and Tables

**Table 1:** Target, mechanism, indication, and status of JAK oral inhibitors for Pso, Psa, AD, and UC.

JAK Inhibitor	Target	Indication	Status
Tofacitinib inhibits ATP binding site	JAK1, JAK2, JAK3, TYK2	PsA UC	FDA/EMA approved FDA/EMA approved
Peficitinib inhibits STAT proteins phosphorylation	JAK1, JAK2, JAK3, TYK2	UC	Discontinued
Baricitinib ATP kinase inhibitor	JAK1, JAK2	AD	FDA/EMA approved
Abrocitinib inhibits ATP binding site	JAK1	AD	FDA/EMA approved
Itacitinib inhibits STAT proteins phosphorylation	JAK1	UC	Phase II
Filgotinib inhibits STAT proteins phosphorylation	JAK1	PsA UC	Phase II FDA/EMA approved
Upadacitinib inhibits STAT proteins phosphorylation	JAK1	PsA UC AD	FDA/EMA approved FDA/EMA approved FDA/EMA approved
Deucravacitinib allosteric inhibitor	TYK2	PSO PsA	FDA/EMA approved Phase II

**Table 2:** Current summary of the clinically approved JAK-STAT inhibitors.

	Tofacitinib	Baricitinib	Abrocitinib	Filgotinib	Upatacitinib	Deucravacitinib
PsA	5 mg b.i.d.				15 mg daily	
PSO						6 mg daily
AD		4 mg daily	200 mg daily		15/30 mg daily	
UC	10 mg b.i.d.for 8 weeks, followed by 5 mg b.i.d.			200 mg daily	45 mg daily for 8 weeks, followed by 15/30 mg daily	

## Data Availability

Not applicable.
